# Enhancing immune protection against MERS-CoV: the synergistic effect of proteolytic cleavage sites and the fusion peptide and RBD domain targeting VLP immunization

**DOI:** 10.3389/fimmu.2023.1201136

**Published:** 2023-05-19

**Authors:** Jeein Oh, Uni Park, Juhyung Kim, Kyeongseok Jeon, Chulwoo Kim, Nam-Hyuk Cho, Youn Soo Choi

**Affiliations:** ^1^ Department of Biomedical Sciences, Seoul National University College of Medicine, Seoul, Republic of Korea; ^2^ Department of Microbiology and Immunology, Seoul National University College of Medicine, Seoul, Republic of Korea; ^3^ Deparatment of Microbiology, Institute for Viral Diseases, Korea University College of Medicine, Seoul, Republic of Korea; ^4^ Seoul National University Bundang Hospital, Seongnam, Gyeonggi-do, Republic of Korea; ^5^ Department of Medicine, Seoul National University College of Medicine, Seoul, Republic of Korea; ^6^ Transplantation Research Institute, Seoul National University Hospital, Seoul, Republic of Korea

**Keywords:** MERS-CoV, PSFP, RBD, virus-like particle (VLP) immunization, spike specific IgG, immune protection

## Abstract

**Introduction:**

The Middle East Respiratory Syndrome Coronavirus (MERS-CoV) is a zoonotic infectious virus that has caused significant outbreaks in the Middle East and beyond. Due to a highly mortality rate, easy transmission, and rapid spread of the MERS-CoV, it remains as a significant public health treat. There is currently no licensed vaccine available to protect against MERS-CoV.

**Methods:**

In this study, we investigated whether the proteolytic cleavage sites and fusion peptide domain of the MERS-CoV spike (S) protein could be a vaccine target to elicit the MERS-CoV S protein-specific antibody responses and confer immune protection against MERS-CoV infection. Our results demonstrate that immunization of the proteolytic cleavage sites and the fusion peptide domain using virus-like particle (VLP) induced the MERS-CoV S protein-specific IgG antibodies with capacity to neutralize pseudotyped MERS-CoV infection in vitro. Moreover, proteolytic cleavage sites and the fusion peptide VLP immunization showed a synergistic effect on the immune protection against MERS-CoV infection elicited by immunization with VLP expressing the receptor binding domain (RBD) of the S protein. Additionally, immune evasion of MERS-CoV RBD variants from anti-RBD sera was significantly controlled by anti-proteolytic cleavage sites and the fusion peptide sera.

**Conclusion and discussion:**

Our study demonstrates the potential of VLP immunization targeting the proteolytic cleavage sites and the fusion peptide and RBD domains of the MERS-CoV S protein for the development of effective treatments and vaccines against MERS-CoV and related variants.

## Introduction

MERS-CoV is a coronavirus that was first identified in Saudi Arabia in 2012 (1) and has caused outbreaks in various countries both in and outside of the Middle East area (2). As of February 2023, 2604 confirmed cases and 936 deaths were reported with the MERS-CoV infection (3). Genetic information of MERS-CoV is translated to express key elements for replication of the viral genome, as well as for the assembly of the virions (2). MERS-CoV also encodes the spike (S) protein, which is expressed on the virus envelope in a trimer form, and comprises S1 and S2 subunits that are essential for virus binding, fusion, and entry into host cells (2, 4).

MERS-CoV infection occurs *via* three key biochemical processes; (i) the protein-protein interaction between the S protein and host cell’s dipeptidyl peptidase IV (DPP4) receptor, (ii) the proteolytic cleavage of the S protein at the S1/S2 and S2’ sites, and (iii) the fusion of the viral envelope with the plasma membrane of the host cells (5, 6). The receptor binding domain (RBD, A368-P586) in the S protein is a specific binding site for the DPP4 molecule (7, 8), making it a prime target for prophylactic MERS-CoV vaccines. In preclinical studies, delivery of the RBD region has shown promise in eliciting RBD-specific humoral and cellular immunity and neutralizing antibodies, as well as in providing immune protection against MERS-CoV infection (9–11).

As a single-stranded RNA virus, MERS-CoV undergoes substantial genetic alterations during replication and transmission stages (12), and these modifications are responsible for the evolution of MERS-CoV, allowing it to infect new hosts and escape the host immune response (13–16). During outbreaks, various mutations have been identified in the RBD region of MERS-CoV strains, with some MERS-CoV variants, including those isolated in South Korea, demonstrating immune evasion from neutralizing antibodies developed against wild-type MERS-CoV or wild-type S protein (14, 17). This finding highlights the potential limitations of a vaccination strategy solely targeting the RBD region and its effectiveness in providing immune protection against MERS-CoV variants.

In this study, we tested whether a middle region of the S2 subunit (E589-F972) covering the proteolytic cleavage sites and the fusion peptide could be targeted to elicit the MERS-CoV S protein-specific antibodies and neutralizing antibodies in mice and could be elaborated with RBD targeting vaccine for synergistic enhancement in immune protection against MERS-CoV and related variants.

## Method

### Mice

C57BL/6J mice and OTII transgenic mice were purchased from DBL (South Korea) and obtained from in-house breeders, respectively. hDPP4 knock-in mice (18) were generously provided by Dr. McCray. All investigations were performed in accordance with the guidelines of Seoul National University Institutional Animal Care and Use Committee (IACUC).

### Plasmids and virus-like particle production

The coding sequences of the headless HA (D18-C59 and C291-Q528) of A/PR/8/1934 (Accession P03452) linked with 4XG, and the receptor binding domain (RBD, A368-P586) and proteolytic cleavage sites and the fusion peptide (E589-F972) regions of the MERS-CoV S protein (Accession YP_009047204) were synthesized and cloned into the CDS position for hen egg lysozyme (HEL) of HEL-GTMCD plasmid (19, 20), an HIV-derived plasmid used to express HEL linked with the transmembrane and cytoplasmic domain of VSV-g protein (**Supplementary Figure 1A**). The HEL-GTMCD and OTII-Hgsyn plasmids were kindly provided by Dr. Vladimir Temchura.

Lentiviral virus-like particles (VLPs) were produced from HEK293T cells by transfecting GTMCD plasmids expressing HEL, headless HA of A/PR/8/1934, or RBD or proteolytic cleavage sites and the fusion peptide region of MERS-CoV with either OTII-Hgsyn or empty Hgsyn plasmid using plain DMEM (Welgene) and polyethylenimine (PEI) (PolyScience). After transfection, HEK293 T cells were cultured in complete DMEM [plain DMEM, 1X Glutamax, 1% antibiotics, 10% FBS], and supernatants were collected daily for 3 days and filtered. Pooled supernatants were ultra-centrifuged at 15,000 rpm for 1.5 hours. The resulting pellets were resuspended, aliquoted with plain DMEM, and stored at -80°C until use.

### VLP quantification

To quantify the amount of VLPs, recombinant HIV1 p24 protein (Abcam) was used as standard (20). VLPs serially diluted in Na_2_CO_3_ coating buffer were coated onto a 96-well plate (Nunc). After overnight incubation, the plate was washed and blocked with 5% PBS-T-F buffer [PBS, 0.05% Tween20, 5% Fat free milk]. The plate was then incubated with anti-p24 antibody (Antibodies-online) followed by anti-mouse IgG-HRP (GeneTex) diluted in 2% PBS-T-F buffer [PBS, 0.05% Tween20, 2% Fat free milk]. The p24 concentration was calculated using the standard curve generated from the recombinant p24 protein.

### Pseudotyped MERS-CoV production

To produce pseudotyped MERS-CoV (pMERS-CoV), HEK293T were transfected with pLP1, pLP2, Luc2P pLVX IRES ZsGreen1, and the MERS-CoV S protein pcDNA3 plasmids. Site-directed mutagenesis using QuickChange lightning site-directed mutagenesis (Agilent) was performed to generate pMERS-CoV harboring D510G and I529T mutant S protein. After transfection, cells were cultured with complete DMEM media. Supernatants were collected daily for three consecutive days and filtered. The supernatants were then ultra-centrifuged at 15,000 rpm for 1.5 hours, and the precipitated pMERS-CoV were resuspended and aliquoted with plain DMEM. The aliquots were stored at -80°C until use.

### Production of hDPP4^+^ HEK293 T cell line

HEK293T cells were transfected with DPP4-pEF-IRES-Puro plasmid and cultured using pre-warmed complete DMEM media with puromycin [plain DMEM, 1X Glutamax, 1 μg/mL antibiotics, 10% FBS]. Expression of hDPP4 was checked using anti-human CD26 APC antibody (BA5b, Biolegend), and hDPP4^+^ HEK293T cells were sorted and subsequently cultured in complete DMEM media with 0.2 μg/mL puromycin.

### Immunization protocol

VLPs that express the headless HA of A/PR/8/1934, HEL, or the RBD or proteolytic cleavage sites and the fusion peptide region of the MERS-CoV were mixed with Addavax (Invivogen) and injected subcutaneously into footpad under anesthesia. To examine CD4 T cell immune responses in an antigen-specific manner, 2.5e5 OTII CD4 T cells, isolated from the spleens of OTII TCRtg mice using Easysep mouse CD4 T cell isolation kit (Stem cell technologies), were adoptively transferred into C57BL/6J mice one day before immunization. For experiments involving booster immunizations, the mice were given VLPs fourteen days after the primary immunization.

### Single cell preparation

Popliteal lymph nodes (popLNs) of VLP-immunized or unimmunized mice were collected in complete DMEM media. Single cell suspensions were obtained after mincing the popLNs on cell strainers (Falcon) using syringe plungers.

### Flow cytometry

The cells were stained with purified or fluorescent-conjugated antibodies against CD4 (RM 4-5), CD19 (6D5), APC-Streptavidin, CD8a (53-6.7), and CD19 (11-26C) from eBioscience; CD4 (RM 4-5), Fas (Jo2), PSGL-1 (2PH1), CD44 (IM7), CD45.1 (A20), CD8a (53-6.7), purified LE/AF rat-anti-mouse CXCR5 (2G8), biotin rat anti-mouse CD138 (281-2), and CD45R (B220) (RA3-6B2) from BD Biosciences; PNA (FL-1071) from Vector Laboratories; PD-1 (J43) from Invitrogen; biotin-SP(long spacer)-conjugated Affipure Goat anti-Rat IgG(H+L) from Jackson Immunoresearch). Three step CXCR5 staining for phenotypic identification of the Tfh cells was performed using purified anti-CXCR5 (2G8) antibody (21).

### Serum antibody ELISA

The mice were bled *via* retro-orbital sinus under anesthesia before immunization or after VLP immunization. The blood was spun at 10,000 rpm for 40 minutes to collect serum. To measure A/PR/8/1934 HA- and MERS-CoV S protein-specific IgGs, a 96-well plate (Nunc) was coated with 0.5 μg/mL of A/PR/8/1934 HA protein (BEI resources) or with 1 μg/mL of the MERS-CoV S protein (Sino Biological) in PBS overnight at 4°C. After wash and blocking with 0.05% PBS-T-B buffer [PBS, 0.05% Tween20, 0.5% BSA], the serum samples, pre-diluted at 1:10 in 0.05% PBS-T-B buffer, were added to top wells, 3-fold serially diluted to the bottom, and incubated for 1.5 hours. After washing, anti-mouse IgG-HRP (Abcam) antibody diluted in 0.1% PBS-T buffer was added and incubated. TMB chromogen solution (Invitrogen) was added for colorimetric development, which was subsequently stopped with stop solution (Invitrogen). O.D. was measured by Sunrise Microplate Reader (Tecan) at a measurement wavelength of 450 nm and a reference wavelength of 570 nm.

### Neutralization assay

Serum samples obtained from mice *via* retro-orbital sinus bleed were stored at -20°C. Prior to performing neutralization assays, the sera were thawed and heat-inactivated at 56°C for 30 minutes. The inactivated serum pre-diluted in 2% DMEM [plain DMEM, 2% FBS] underwent 4-fold serial dilution. Pseudotyped MERS-CoV was added to the mixture and incubated in 4°C cold room for 1 hour on a rotator. The mixture was then added to hDPP4+ HEK293T cells on a 96-well plate. After centrifugation of the plate at 1,500 rpm for 1 hour at 25°C, the media was replaced with 10% DMEM media [plain DMEM, 10% FBS]. After 2 days of culture, the cells were washed with PBS and lysed with cell culture lysis buffer (Promega) and centrifuged at 2,000 rpm for 5 minutes. The supernatant was transferred to a 96-well white plate (SPL) with luciferase assay reagent (Promega). The luminescence was measured using a microplate reader (Tecan) with following conditions: wait time of 2 seconds and integration time of 1000 milliseconds.

### Viral infection studies

C57BL/6J mice were subcutaneously immunized with VLPs expressing the headless HA of A/PR/8/1934 or VLPs expressing HEL on day 0 and day 14. Ten days after the booster immunization, the mice were intranasally infected with 150 plaque-forming units (PFU) of A/PR/8/1934. hDPP4-Tg mice were subcutaneously immunized with VLPs expressing the proteolytic cleavage sites and the fusion peptide region of MERS-CoV or HEL on day 0 and day 14. On day 21, VLPs expressing the RBD region of MERS-CoV were administered to the immunized mice. Seven days later, both the immunized and unimmunized groups of mice were intranasally infected with 10^4^ PFU of mouse adapted MERS-CoV (18). A/PR/8/1934 and mouse adapted MERS-CoV were generously provided by Drs. Nicole Baumgarth and Paul McCray Jr. respectively.

### Statistical analysis

Either paired or unpaired student’s t-test and one-way ANOVA with Tukey’s multiple comparison test were used for statistical analyses using GraphPad Prism 7. P-values less than 0.05 were considered statistically significan (*: p<0.05, **: p<0.01, ***: p<0.001, ****: p < 0.0001).

## Results

### VLP mediated antigen delivery elicits immune protection against respiratory viral infection

Virus-like particle (VLP) is an effective platform to deliver target antigens in vaccines due to its similar morphologic features to infectious viruses while lacking viral genome required for replication (22). As a proof-of-concept study for eliciting immune protection against respiratory viral infection by a VLP immunization system, we first tested whether this system could induce target antigen-specific antibody responses and might provide immune protection using a mouse model of influenza A virus (IAV) infection.

**Figure 1:**
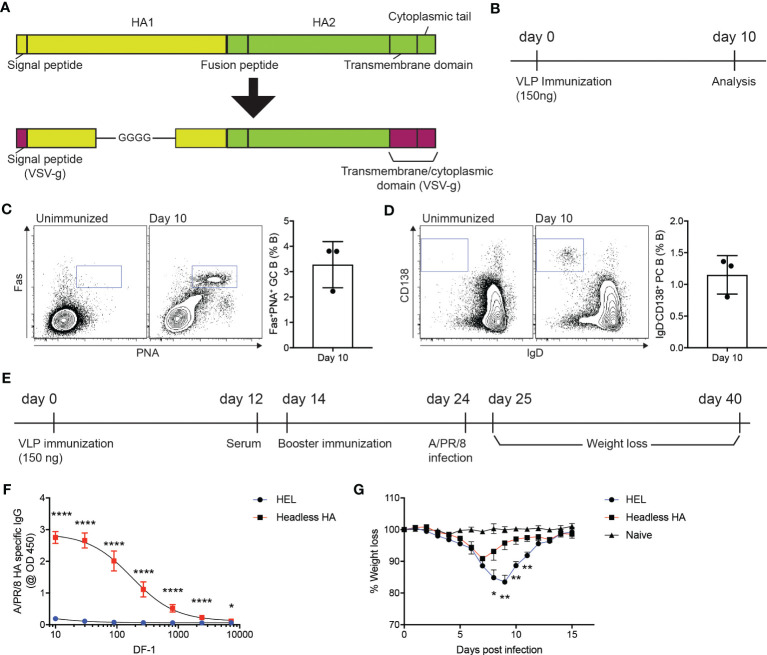
VLP-mediated delivery of HA antigen confers immune protection against influenza virus infection. **(A)** Schematic diagram of the headless hemagglutinin (HA) expressed on lentiviral virus-like particles (VLPs). The signal peptide and the transmembrane and cytoplasmic domains of the VSV-g protein are linked with the HA region lacking the globular head domain at the N- and C-terminus, respectively. **(B–D)**. **(B)** Experimental scheme. C57BL/6J mice were immunized with headless HA-expressing VLPs emulsified with Addavax on day 0. Ten days after immunization, popliteal lymph nodes (popLNs) were analyzed for germinal center formation and plasma cell differentiation. **(C, D)** Shown are FACS plots of B cells in the popLNs of the immunized mice and an unimmunized mouse. Gates indicate FAS^+^PNA^+^ germinal center (GC) B cells and IgD^-^CD138^+^ plasma cells (PCs), respectively. Shown are representatives of two independent experiments (three mice per experiment). **(E–G)**. **(E)** C57BL/6J mice were immunized with VLPs expressing the headless HA or HEL on days 0 and 14. On day 24, the mice were intranasally infected with A/PR/8/1934. The infected and uninfected (naïve) mice were monitored for weight loss for the next 14 days. **(F)** The immunized mice were bled on day 12. HA-specific IgG levels were measured. **(G)** Shown are weight loss of the infected and uninfected mice. Composite data from two independent experiments (n=7 mice for the immunized group; n=6 for the unimmunized group). Data were analyzed using unpaired student’s t-test **(F)** and one-way ANOVA with Tukey’s multiple comparison **(G)**. *p < 0.05, **p < 0.01, ****p < 0.0001.

Influenza virus infects host cells *via* binding of the hemagglutinin (HA) protein to sialic acids present on the glycoproteins in the respiratory tract (23), which led the whole or part of the HA protein to be targeted in vaccines (24). The coding sequence for the HA region of A/PR/8/1934 strain that lacks globular head domain (D18-C59-GGGG-C291-Q528) (25) was synthesized and subsequently cloned into GTMCD plasmid to produce lentiviral VLPs expressing the headless HA (headless HA VLP hereafter) (**Figure 1A**). We then examined whether immunization of the headless HA VLP could trigger humoral immune responses in mice. Ten days after subcutaneous immunization of the headless HA VLP, popliteal lymph nodes (popLNs) were analyzed for germinal center (GC) formation and plasma cell (PC) differentiation of B cells (**Figure 1B**). We found Fas^+^PNA^+^ GC B cells and IgD^-^CD138^+^ PC B cells formed robustly in the popLNs after the headless HA VLP immunization (**Figures 1C, D**), indicating that the headless HA VLP immunization could induce the production of HA specific antibodies and might contribute to immune protection against A/PR/8/1934 infection.

C57BL/6J mice were immunized with the headless HA VLP or with VLPs that express hen egg lysozyme (HEL) as a negative control (**Figure 1E**). Twelve days after the primary immunization, the immunized mice were bled to measure A/PR/8/1934 HA-specific IgG antibodies (**Figure 1E**). As expected, HA-specific IgG antibodies were strongly induced in mice immunized with the headless HA VLP compared to the mice immunized with VLPs expressing HEL (**Figure 1F**). The mice were given with a booster immunization on day 14 and were intranasally infected with A/PR/8/1934 on day 24. We monitored the body weight of the mice for the next fourteen days to examine whether the headless HA VLP immunization contributed to the immune protection against IAV infection (**Figure 1E**). The mice immunized with the headless HA VLP lost body weight significantly less and began to gain weight two days earlier than the control mice (**Figure 1G**). These data indicate that the immunogenicity of the antigen of interest could be assessed using VLP-mediated antigen delivery system.

### Proteolytic cleavage sites and the fusion peptide targeting VLP immunization induces MERS-CoV spike protein specific antibody responses

**Figure 2:**
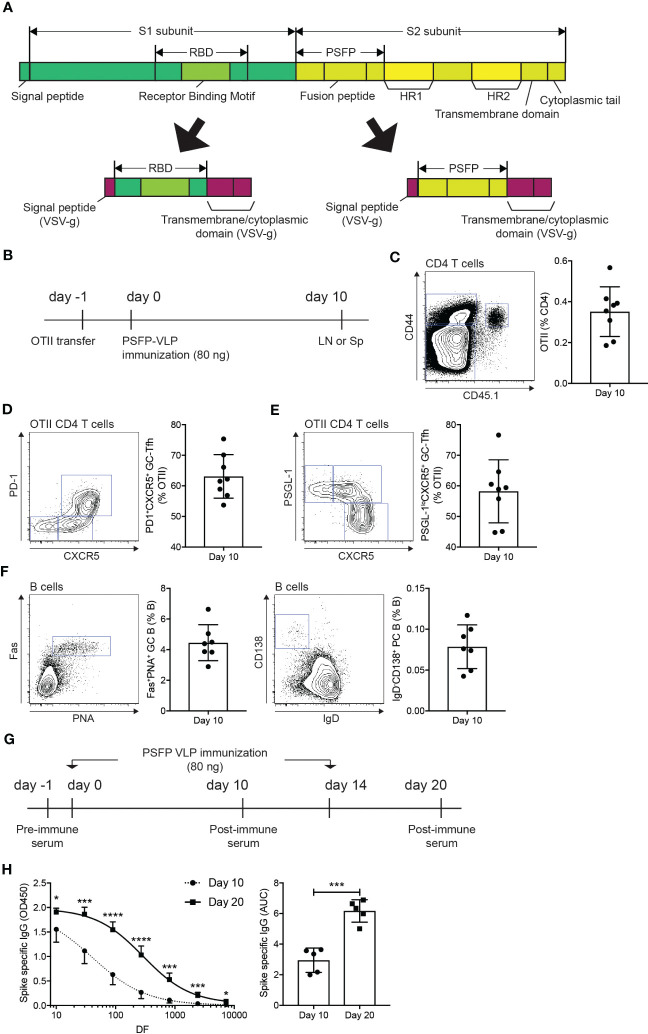
Immunization of VLPs expressing MERS-CoV proteolytic cleavage sites and the fusion peptide leads to the MERS-CoV spike protein specific humoral immunity. **(A)** Schematic diagrams of the spike protein of MERS-CoV and the RBD and proteolytic cleavage sites and the fusion peptide (PSFP) regions expressed on the surface of VLPs. **(B–F)**. **(B)** Experimental scheme. A day before immunization, CD45.1^+^ OTII TCRtg CD4 T cells were adoptively transferred into CD45.2^+^ C57BL/6J mice. On day 0, the mice were subcutaneously immunized with VLP expressing the MERS-CoV proteolytic cleavage sites and the fusion peptide (PSFP). popLNs were examined for OTII CD4 T cell responses and B cell responses on day 10. **(C)** FACS plot of CD4 T cells in the popLNs with gate indicating CD45.1^+^ CD44^hi^ OTII CD4 T cells. The OTII CD4 T cell frequency among total CD4 T cells. **(D, E)** OTII CD4 T cells were analyzed for Tfh differentiation. Shown are FACS plots of OTII CD4 T cells with gates indicating PD-1^+^CXCR5^+^ **(D)** and PSGL-1^lo^CXCR5^+^ **(E)** germinal center Tfh cells. The frequencies of the respective cells among total OTII CD4 T cells. **(F)** Shown are FACS plots of B cells. Gates indicate FAS^+^PNA^+^ GC B cells and IgD^-^CD138^+^ PCs, respectively. Frequencies of each population among total B cells. Composite data from two independent experiments were analyzed (eight and seven mice for the investigations of OTII CD4 T cells and B cells, respectively). **(G, H)**. **(G)** C57BL/6J mice were subcutaneously immunized with VLPs expressing the MERS-CoV proteolytic cleavage sites and the fusion peptide (PSFP) on days 0 and 14. **(H)** On days 10 and 20, the mice were bled to measure the MERS-CoV S protein specific IgG. Area under curves (AUCs) were calculated. Shown are a representative of two independent experiments using 5 mice. Statistical analyses were performed using unpaired student’s t-test. *p < 0.05, ***p < 0.001, ****p < 0.0001.

Studies have shown that the S protein of MERS-CoV undergoes a conformational change through proteolytic cleavages, resulting in the exposure of the fusion peptide domain, which allows the virus to fuse with the plasma membrane of the host cells (26). Therefore, we considered whether antibody responses against the cleavage sites and the fusion peptide domain could potentially contribute to immune protection against MERS-CoV infection. To address this point, we cloned the coding sequence of the amino acid region (E589-F972) of the S2 subunit, which covers proteolytic cleavage sites (S1/S2 interface and S2’ position) and the fusion peptide, into the G-TMCD plasmid (**Figure 2A**). Using OTII-Hgsyn plasmid (27), we produced lentiviral VLPs that express the proteolytic cleavage sites and the fusion peptide domain of the MERS-CoV S protein on the surface and OTII peptide (OVA323-339) linked to the C-terminus of the HIV matrix protein. After immunization of VLPs expressing the MERS-CoV proteolytic cleavage sites and the fusion peptide and OTII peptide, immune responses of CD4 T cells could be examined in an antigen specific manner using OTII TCRtg (specific for OTII peptide displayed on I-Ab) CD4 T cells. CD45.1^+^ OTII CD4 T cells were adoptively transferred into C57BL/6J mice, which were subsequently immunized with VLPs expressing the MERS-CoV proteolytic cleavage sites and the fusion peptide and OTII peptide (**Figure 2B**). Immune responses of B cells and antigen specific CD4 T cells in the popLNs were examined ten days after immunization. We found that the VLP immunization led to a robust expansion (**Figure 2C**) and follicular helper CD4 T cell differentiation (**Figures 2D, E**) of OTII CD4 T cells. It indicates that CD4 T cell dependent humoral immunity could be strongly elicited by the VLP immunization. Indeed, Fas^+^PNA^+^ GC B cells and IgD^-^CD138^+^ PC B cells were readily identified in the popLNs after immunization of VLPs expressing the MERS-CoV proteolytic cleavage sites and the fusion peptide and OTII peptide (**Figure 2F**).

Strong Tfh differentiation and GC and PC B cell development in the lymph node following the VLP immunization implied that the administration of the MERS-CoV proteolytic cleavage sites and the fusion peptide domain *via* VLP could vigorously induce S protein specific antibodies in the mice. C57BL/6J mice, immunized with VLPs expressing the MERS-CoV proteolytic cleavage sites and the fusion peptide and OTII peptide on days 0 and 14, were bled to measure the S protein specific IgG antibodies in the serum after the primary and booster immunization (**Figure 2G**). S protein specific IgGs were nicely induced by the primary immunization, which was further enhanced when the mice were given with a booster immunization (**Figure 2H**).

### Anti-MERS-CoV immunity can be enhanced by VLP immunization targeting RBD and proteolytic cleavage sites and the fusion peptide

**Figure 3:**
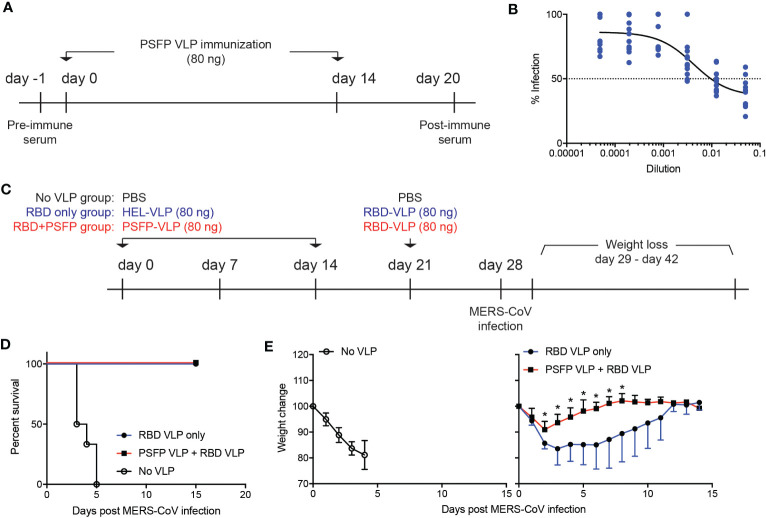
Targeting proteolytic cleavage sites and the fusion peptide in addition to RBD in VLP immunization can enhance immune protection against MERS-CoV infection. **(A–B)**. **(A)** Experimental scheme. C57BL/6J mice were subcutaneously immunized with VLPs expressing the MERS-CoV proteolytic cleavage sites and the fusion peptide (PSFP) on days 0 and 14. Anti-sera was obtained from the mice on day 20 for microneutralization assay. **(B)** Shown are relative luciferase activities of hDPP4^+^ HEK293T cells infected with pMERS-CoV in the presence of anti-sera from the immunized mice (n=11) compared to that of hDPP4^+^ HEK293T cells infected with pMERS-CoV. Dotted line indicates inhibition of pMEFS-CoV entry into hDPP4^+^ HEK293T cells by 50% (IC50) in the presence of the immune sera. **(C–E)**. **(C)** Experimental scheme. C57BL/6J mice were subcutaneously immunized with VLPs expressing MERS-CoV proteolytic cleavage sites and the fusion peptide (PSFP) or HEL on days 0 and 14. On day 21, the mice were subsequently immunized with VLPs expressing MERS-CoV RBD. On day 28, the immunized and unimmunized (PBS treated) mice were intranasally infected with maMERS-CoV. Survival and body weight of the mice were monitored for the next 14 days. **(D)** Survival of the mice after MERS-CoV infection. **(E)** Changes in the body weight of the mice after infection. Composite date from two independent experiments (n=5 for the immunized, n=6 for the unimmunized). Unpaired student’s t-test comparisons were used for statistical analysis. *p < 0.05.

Immune protection against MERS-CoV is contributed by neutralizing antibodies that inhibit viral entry into host cells. As VLPs expressing the MERS-CoV proteolytic cleavage sites and the fusion peptide robustly induced the S protein specific antibodies (**Figure 2H**), we then explored whether these antibodies might exhibit neutralizing activity against MERS-CoV. C57BL/6J mice were immunized with VLPs expressing the MERS-CoV proteolytic cleavage sites and the fusion peptide and OTII peptide (days 0 and 14), whose serum was collected six days after the booster immunization (**Figure 3A**). hDPP4 expressing HEK293T cells and pseudotyped MERS-CoV (pMERS-CoV) that express the MERS-CoV S protein (**Supplementary Figures 1B, C**) were used to assess the neutralization activity of the serum. The dilution of the sera that inhibit 50% of pMERS-CoV infection was between 9/1000 and 1/100 (**Figure 3B**), which indicates that the VLP immunization could generate neutralizing antibodies against MERS-CoV in mice.

We then investigated whether proteolytic cleavage sites and the fusion peptide targeting immunization could confer immune protection against MERS-CoV infection. To address this point, we immunized hDPP4 expressing mice with VLPs that express either the MERS-CoV proteolytic cleavage sites and the fusion peptide or HEL, an irrelevant antigen, on days 0 and 14. On day 21, the mice were given with VLPs that express the receptor binding domain (RBD) (**Figure 2A**) of the MERS-CoV S protein (**Figure 3C**). Seven days after immunization with VLPs expressing the MERS-CoV RBD, the mice were intranasally infected with mouse-adapted MERS-CoV (maMERS-CoV). As a negative control, unimmunized hDPP4 expressing mice were infected with maMERS-CoV. The infected mice were monitored for the next 14 days (**Figure 3C**). We found that all mice in the immunized groups survived, whereas the unimmunized mice succumbed to maMERS-CoV infection (**Figure 3D**), which strongly implies that the VLP immunization successfully elicited protective immunity against MERS-CoV. Moreover, the mice immunized with both the proteolytic cleavage sites and the fusion peptide expressing VLPs and the RBD expressing VLPs lost significantly less body weight than the mice given the RBD expressing VLPs alone (**Figure 3E**). Hence, our data suggest that immune protection against MERS-CoV could be augmented using vaccines that target the RBD and proteolytic cleavage sites and the fusion peptide regions of the S protein of the MERS-CoV.

### The ability of MERS-CoV RBD variants to evade immune response induced by RBD-targeting vaccines can be limited by the presence of anti-proteolytic cleavage sites and the fusion peptide antibodies

RBD-targeting immunizations have been reported to provide immune protection against MERS-CoV infection by generating neutralizing antibodies (8, 11). Antibodies elicited against the RBD region of wild-type MERS-CoV, however, have been shown to lose their neutralizing capacity against MERS-CoV mutants that harbor amino acid changes in the RBD region (17). Relatively few mutations observed in the proteolytic cleavage sites and the fusion peptide region of MERS-CoV isolates compared to the RBD region (**Supplementary Table 1**) led us to speculate that anti-proteolytic cleavage sites and the fusion peptide antibodies may provide immune protection against MERS-CoV variants that have acquired mutations in the RBD region.

**Figure 4:**
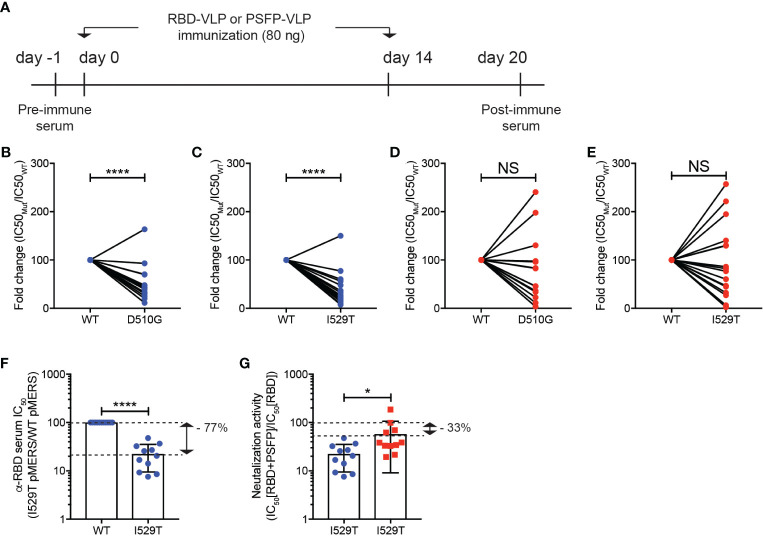
Immune evasion of MERS-CoV RBD variants from RBD targeting vaccination could be inhibited by immunization of VLPs expressing the MERS-CoV proteolytic cleavage sites and the fusion peptide. **(A–E)**. **(A)** Experimental scheme. C57BL/6J mice were immunized with VLPs expressing RBD or proteolytic cleavage sites and the fusion peptide (PSFP) of wild-type MERS-CoV on days 0 and 14. The anti-sera were obtained from the mice on day 20. **(B–E)** Shown are fold changes in IC50 of the anti-RBD **(B, C)** and anti- proteolytic cleavage sites and the fusion peptide (PSFP) **(D, E)** sera against wild-type pMERS-CoV over that against D510G **(B, D)** and I529T **(C, E)** pMERS-CoV. The anti-RBD and anti-proteolytic cleavage sites and the fusion peptide (PSFP) sera were collected from 17 and 14 mice immunized with the corresponding VLP, respectively. **(F, G)** The anti-RBD sera, after mixed with either pre- **(F)** or post-immune **(G)** sera of the mice immunized with VLPs expressing the wild-type MERS-CoV proteolytic cleavage sites and the fusion peptide (PSFP) were used for neutralization assays using wild-type or I529T pMERS-CoV. The sera were obtained from 11 mice immunized with VLPs expressing RBD or proteolytic cleavage sites and the fusion peptide (PSFP) of wild-type MERS-CoV. Unpaired student’s t-test comparisons were used for statistical analysis. *p < 0.05, ****p < 0.0001; NS, non-statistically significant.

To test our hypothesis, we generated pMERS-CoV expressing the S protein with D510G or I529T amino acid substitutions, which were found in MERS-CoV variants isolated during the outbreak in South Korea (12, 14). Both D510G and I529T pMERS-CoV were shown to infect hDPP4 expressing HEK293T cells as robustly as, or more strongly than, the wild-type pMERS-CoV (**Supplementary Figures 1D, E**). Sera against the unmutated RBD or proteolytic cleavage sites and the fusion peptide regions were obtained from the mice immunized with VLPs expressing RBD or proteolytic cleavage sites and the fusion peptide of the wild-type MERS-CoV (**Figure 4A**). As reported previously, the anti-RBD sera significantly lost neutralizing capacity against D510G pMERS-CoV compared to that against wild-type pMERS-CoV (**Figure 4B**), while it strongly bound to the S protein of the wild-type MERS-CoV (**Supplementary Figure 1F**). Moreover, the neutralizing ability of the anti-RBD sera was further reduced against I529T pMERS-CoV (**Figure 4C**). In contrast, the anti-proteolytic cleavage sites and the fusion peptide sera exhibited modest loss in neutralizing activity against D510G pMERS-CoV and I529T pMERS-CoV, about 20 and 10% of that against wild-type pMERS-CoV, respectively (**Figures 4D, E**).

A synergy in immune protection against MERS-CoV infection by targeting both RBD and proteolytic cleavage sites and the fusion peptide in the VLP immunization (**Figure 3E**) led us to speculate that the ability of MERS-CoV RBD variants to evade neutralizing activity of anti-RBD sera might be limited in the presence of anti-proteolytic cleavage sites and the fusion peptide antibodies. To test this hypothesis, we quantified the luciferase activity of hDPP4^+^ HEK293T cells after infection with either wild-type or I529T pMERS-CoV in the presence of the anti-RBD sera mixed 1-to-1 with the pooled sera of pre-immune mice or mice immunized with VLPs expressing wild type proteolytic cleavage sites and the fusion peptide (**Figure 4A**). In the presence of pre-immune serum, the anti-RBD sera lost neutralizing activity against I529T pMERS-CoV (about 77% of that against wild-type pMERS-CoV) (**Figure 4F**). Interestingly, we found that the neutralizing activity of the anti-RBD sera against I529T pMERS-CoV increased when the anti-proteolytic cleavage sites and the fusion peptide sera was added. Relative comparisons of the neutralizing activity of the “anti-RBD + anti-proteolytic cleavage sites and the fusion peptide” sera with that of the “anti-RBD + pre-immune mixture” against I529T pMERS-CoV revealed the neutralizing capacity of the anti-RBD sera restored about 45% in the presence of the anti-proteolytic cleavage sites and the fusion peptide sera (**Figure 4G**). Collectively, these data strongly imply that MERS-CoV RBD variant’s immune escape could be limited by combination immunization targeting the RBD and proteolytic cleavage sites and the fusion peptide region of the S protein.

## Discussion

MERS-CoV has been around in human community for more than a decade, yet vaccines or therapeutics against MERS-CoV are not available. In this study, we investigated the potential of lentiviral VLP immunization against the proteolytic cleavage sites and fusion peptide domain of the MERS-CoV S protein, in combination with RBD domain targeting immunization, for the development of effective treatments and vaccines against MERS-CoV and related variants. Our results showed that immunization of VLPs expressing the proteolytic cleavage sites and the fusion peptide region of MERS-CoV induced the MERS-CoV S-specific IgG antibodies with neutralizing capacity and further enhanced the immune protection against MERS-CoV elicited by immunization with RBD targeting VLPs. Moreover, our study demonstrated the proteolytic cleavage sites and the fusion peptide domain as an ideal complementary target to vaccines against the RBD domain for controlling the immune evasion of MERS-CoV RBD variants.

MERS-CoV infection occurs *via* fusion with the plasma membrane of host cells after proteolytic cleavage of the S protein that leads to the fusion peptide accessible for fusion process (28). One might thus expect that MERS-CoV infection might be controlled by inhibiting proteolytic cleavages of the MERS-CoV S protein and by impeding fusion peptide function. This speculation is supported by studies that demonstrate anti-viral activities of E-64-D and Camostat, inhibitors of Cathepsin-L and TMPRSS2, respectively (5, 6, 29), and of fusion inhibitor peptides (30, 31). Entry of pseudotyped MERS-CoV and MERS-CoV into airway epithelial cells or DPP4 expressing cells and plaque formation of MERS-CoV were severely inhibited by reduced activity and function of the proteases and fusion peptide. Our study suggest that this strategy might also be accomplished by vaccination that elicits antibodies responses against the sites where Cathepsin-L and TMPRSS2 recognize for cleavages of the S protein and the fusion peptide that interacts with the DPP4 receptor for triggering fusion process.

Broadly neutralizing antibodies (bnAbs) that recognize highly conserved epitopes of viruses can neutralize various strains and have been identified in a small group of patients infected with HIV-1 and IAV (32, 33). The amino acid sequence of the proteolytic cleavage sites and the fusion peptide region (E589-F972) is conserved by the wild-type MERS-CoV and the D510G and I529T MERS-CoV RBD variants (**Supplementary Table 1**). This suggests that the proteolytic cleavage sites and the fusion peptide region of MERS-CoV might be targeted to generate bnAbs against MERS-CoV and its related variants in vaccines. B cell clones that are specific for the 887RSAIEDLLF895 epitope of the proteolytic cleavage sites and the fusion peptide region (E589-F972) might provide cross-reactive neutralizing antibodies against SARS-CoV-2 and variants of concern, given that bnAbs, discovered in COVID-19 convalescent patients and in vaccinated individuals previously infected with SARS-CoV-2, exhibited epitope specificities of 815RSFIEDLLF823 and K811PSKRSFIEDLLFNK825, the region encompassing S2’ cleavage site and the fusion peptide of the SARS-CoV-2 S protein (34, 35). It would be thus interesting to investigate whether sequential immunization with VLPs expressing MERS-CoV proteolytic cleavage sites and the fusion peptide and VLPs expressing the region exhibiting high sequence homology in SARS-CoV, MERS-CoV, and SARS-CoV-2 could elicit bnAbs against human-infecting coronaviruses.

## Data availability statement

The original contributions presented in the study are included in the article/**Supplementary Material**, further inquiries can be directed to the corresponding author/s.

## Ethics statement

The animal study was reviewed and approved by Seoul National University College of Medicine (SNUCM) and Seoul National University Hospital (SNUH).

## Author contributions

Conceptualization: YSC. Methodology: JO, JK, CK, N-HC, and YSC. Investigation: JO, JK, UP, KJ, N-HC, and YSC. Funding acquisition: NC and YSC. Project administration: YSC. Supervision: YSC. Writing: YSC. All authors contributed to the article and approved the submitted version.

## Supplementary material

Supplementary Figure 1 Infection of hDPP4^+^ HEK293T cells with pMERS-CoVs and the MERS-CoV S protein specific IgG production by immunization of VLPs expressing MERS-CoV RBD. **(A)** Map of the HEL-GTMCD plasmid that express HEL linked with the signal peptide and the transmembrane and cytoplasmic domain of VSV-g protein. **(B)** hDPP4 histograms of HEK293T cells transfected with empty or hDPP4 expressing plasmids. **(C–E)** Luciferase activities of hDPP4^+^ HEK293T cells infected with **(C)** wild-type pMERS-CoV, **(D)** D510G pMERS-CoV, or **(E)** I529T pMERS-CoV. **(F)** C57BL/6J mice were immunized with VLPs expressing unmutated MERS-CoV RBD on days 0 and 14. The mice were bled on days 10 and 20 to measure the S protein specific IgG. Shown are representative of two independent experiments using 5 mice. Unpaired student’s t-test was used to determine statistical values. *p < 0.05; **p < 0.01; ***p < 0.001.

Supplementary Table 1 The amino acid sequences of the proteolytic cleavage sites and the fusion peptide region of the wild-type MERS-CoV and the D510G and I529T MERS-CoV variants.
