# Supplementary figures and images for: Enhancing immune protection against MERS-CoV: the synergistic effect of proteolytic cleavage sites and the fusion peptide and RBD domain targeting VLP immunization
Source: Front Immunol. 2023 May 19;14:1201136. doi: 10.3389/fimmu.2023.1201136 (PMC10235442; doi:10.3389/fimmu.2023.1201136)

**A**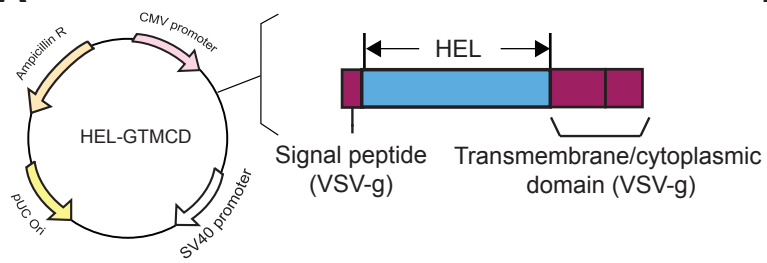**B**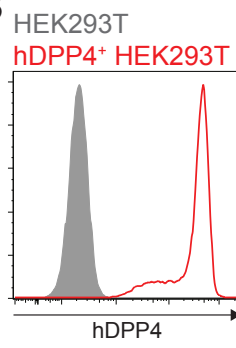**C**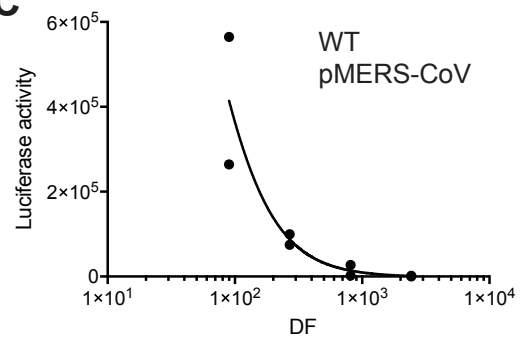**D**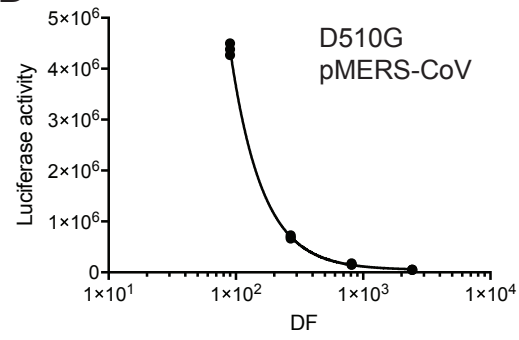**E**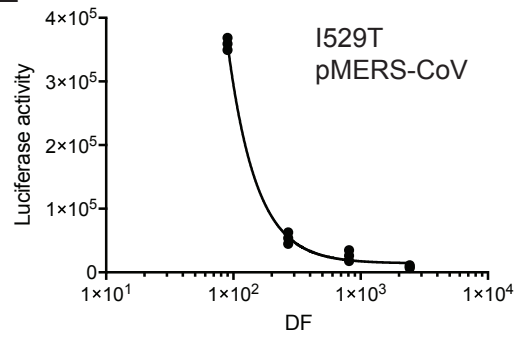**F**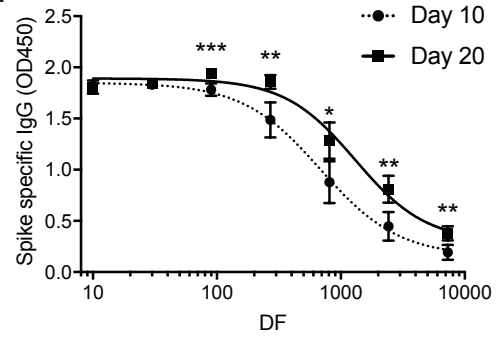

Supplement: Supplementary Figure 1 — Infection of hDPP4+ HEK293T cells with pMERS-CoVs and the MERS-CoV S protein specific IgG production by immunization of VLPs expressing MERS-CoV RBD. (A) Map of the HEL-GTMCD plasmid that express HEL linked with the signal peptide and the transmembrane and cytoplasmic domain of VSV-g protein. (B) hDPP4 histograms of HEK293T cells transfected with empty or hDPP4 expressing plasmids. (C–E) Luciferase activities of hDPP4+ HEK293T cells infected with (C) wild-type pMERS-CoV, (D) D510G pMERS-CoV, or (E) I529T pMERS-CoV. (F) C57BL/6J mice were immunized with VLPs expressing unmutated MERS-CoV RBD on days 0 and 14. The mice were bled on days 10 and 20 to measure the S protein specific IgG. Shown are representative of two independent experiments using 5 mice. Unpaired student’s t-test was used to determine statistical values. *p < 0.05; **p < 0.01; ***p < 0.001. [file DataSheet_2.pdf]
