# Supplementary material for: Enhancing immune protection against MERS-CoV: the synergistic effect of proteolytic cleavage sites and the fusion peptide and RBD domain targeting VLP immunization
Source: Front Immunol. 2023 May 19;14:1201136. doi: 10.3389/fimmu.2023.1201136 (PMC10235442; doi:10.3389/fimmu.2023.1201136)
Supplement: Supplementary Table 1 — The amino acid sequences of the proteolytic cleavage sites and the fusion peptide region of the wild-type MERS-CoV and the D510G and I529T MERS-CoV variants. [file DataSheet_1.pdf]

#	Accession number	Mutation	RBD (368-586)	PSFP (589-972)	Other region	
1	JX869059	-	-	-	-	
2	JX869059.2	+	-	-	+	
3	KC164505.2	+	+	-	-	
4	KC667074.1	+	+	-	-	
5	KC776174.1	+	-	-	+	
6	KF192507.1	-	-	-	-	
7	KF600612.1	+	+	-	-	
8	KF600613.1	-	-	-	-	
9	KF600628.1	+	-	-	+	
10	KF600630.1	+	-	-	+	
11	KF600652.1	-	-	-	-	
12	KF745068.1	+	-	+	+	Q914H
13	KF811036.1	+	-	-	+	
14	KF958702.1	-	-	-	-	
15	KF961221.1	+	+	-	-	
16	KF961222.1	+	+	-	-	
17	KJ156869.1	+	+	-	-	
18	KJ156873.1	-	-	-	-	
19	KJ156874.1	+	-	-	+	
20	KJ156876.1	+	-	-	+	
21	KJ156881.1	+	-	-	+	
22	KJ156901.1	+	-	-	+	
23	KJ156905.1	+	-	-	+	
24	KJ156910.1	+	-	-	+	
25	KJ156911.1	+	-	-	+	
26	KJ156920.1	+	-	-	+	
27	KJ156934.1	-	-	-	-	
28	KJ156936.1	+	-	-	+	
29	KJ156944.1	+	-	-	+	
30	KJ156949.1	-	-	-	-	
31	KJ156952.1	-	-	-	-	
32	KJ361500.1	+	-	+	+	Q914H
33	KJ361501.1	+	-	+	+	Q914H
34	KJ361503.1	+	-	+	+	Q914H
35	KJ556336.1	-	-	-	-	
36	KM015348.1	-	-	-	-	
37	KM027277.1	+	+	-	-	
38	KM027279.1	+	+	-	+	
39	KM027281.1	+	-	-	+	
40	KM027284.1	+	+	-	+	
41	KM027285.1	+	-	-	+	
42	KM027286.1	+	-	-	+	
43	KM210277.1	-	-	-	-	
44	KM210278.1	-	-	-	-	
45	KP209312.1	+	-	-	+	
46	KP236092.1	+	-	-	+	
47	KR011263.1	-	-	-	-	
48	KR011264.1	-	-	-	-	
49	KR011265.1	-	-	-	-	
50	KR011266.1	-	-	-	-	
51	KT006149.2	-	-	-	-	
52	KT026453.1	-	-	-	-	
53	KT026454.1	-	-	-	-	
54	KT029139.1	-	-	-	-	
55	KT182953	-	-	-	-	
56	KT182954	+	+	-	-	I529T
57	KT182955	+	+	-	-	I529T
58	KT182956	+	+	-	-	I529T
59	KT182957	+	+	-	-	I529T

	60	KT182958	+	-	-	+	
Park et al. 2016 Cold Spring Harb Mol Case Stud	61	KX034093.1	+	+	-	+	I529T
	62	KX034094.1	+	+	-	+	D510G
	63	KX034095.1	+	+	-	+	D510G
	64	KX034096.1	+	+	-	+	I529T
	65	KX034097.1	+	+	-	+	D510G
	66	KX034098.1	+	+	-	+	I529T
	67	KX034099.1	+	+	-	+	I529T
	68	KX034100.1	+	+	-	+	I529T
Kim et al. 2016. mBio	69	KT868877.1	+	+	-	+	I529T
	70	KT868876.1	+	+	-	+	I529T
	71	KT868875.1	+	+	-	+	D510G
	72	KT868874.1	+	+	-	+	I529T
	73	KT868873.1	+	+	-	+	I529T
	74	KT868872.1	+	+	-	+	I529T
	75	KT868871.1	+	+	-	+	I529T
	76	KT868870.1	+	-	-	+	
	77	KT868869.1	+	+	-	+	I529T
	78	KT868868.1	+	+	-	+	I529T
	79	KT868867.1	+	+	-	+	I529T
	80	KT868866.1	+	+	-	+	I529T
	81	KT868865.1	+	+	-	+	I529T
	82	KT036372.1	+	-	-	+	
Chung et al. 2018.	83	MH978886.1	+	-	-	+	
	84	MH978887.1	+	-	-	+	
	85	MH978888.1	+	-	-	+	

	Mutation	RBD (368-586)	PSFP (589-972)	Other region
Total isolates	85			
Isolates with mutations	63	33	4	26
%	74.12	52.38	6.35	41.27
